# No evidence for involvement of *SDHD *in neuroblastoma pathogenesis

**DOI:** 10.1186/1471-2407-4-55

**Published:** 2004-08-24

**Authors:** Katleen De Preter, Jo Vandesompele, Jasmien Hoebeeck, Caroline Vandenbroecke, Jöel Smet, Annick Nuyts, Geneviève Laureys, Valérie Combaret, Nadine Van Roy, Frank Roels, Rudy Van Coster, Marleen Praet, Anne De Paepe, Frank Speleman

**Affiliations:** 1 Center for Medical Genetics, Ghent University Hospital, K5, De Pintelaan 185, B-9000 Ghent, Belgium; 2 Department of Pathological Anatomy, Ghent University Hospital, BLOK A, De Pintelaan 185, B-9000 Ghent, Belgium; 3 Department of Paediatrics, Ghent University Hospital, K6, De Pintelaan 185, B-9000 Ghent, Belgium; 4 Molecular Oncology Unit, Centre Léon Bérard, 28 rue Laennec, F-69373 Lyon, France

## Abstract

**Background:**

Deletions in the long arm of chromosome 11 are observed in a subgroup of advanced stage neuroblastomas with poor outcome. The deleted region harbours the tumour suppressor gene *SDHD *that is frequently mutated in paraganglioma and pheochromocytoma, which are, like neuroblastoma, tumours originating from the neural crest. In this study, we sought for evidence for involvement of *SDHD *in neuroblastoma.

**Methods:**

*SDHD *was investigated on the genome, transcriptome and proteome level using mutation screening, methylation specific PCR, real-time quantitative PCR based homozygous deletion screening and mRNA expression profiling, immunoblotting, functional protein analysis and ultrastructural imaging of the mitochondria.

**Results:**

Analysis at the genomic level of 67 tumour samples and 37 cell lines revealed at least 2 bona-fide mutations in cell lines without allelic loss at 11q23: a 4bp-deletion causing skip of exon 3 resulting in a premature stop codon in cell line N206, and a Y93C mutation in cell line NMB located in a region affected by germline *SDHD *mutations causing hereditary paraganglioma. No evidence for hypermethylation of the *SDHD *promotor region was observed, nor could we detect homozygous deletions. Interestingly, *SDHD *mRNA expression was significantly reduced in *SDHD *mutated cell lines and cell lines with 11q allelic loss as compared to both cell lines without 11q allelic loss and normal foetal neuroblast cells. However, protein analyses and assessment of mitochondrial morphology presently do not provide clues as to the possible effect of reduced *SDHD *expression on the neuroblastoma tumour phenotype.

**Conclusions:**

Our study provides no indications for 2-hit involvement of *SDHD *in the pathogenesis of neuroblastoma. Also, although a haplo-insufficient mechanism for *SDHD *involvement in advanced stage neuroblastoma could be considered, the present data do not provide consistent evidence for this hypothesis.

## Background

Neuroblastoma (NB) is the most frequent extra-cranial solid tumour in children, originating from immature neural crest cells of the sympathetic nervous system [1]. The tumours show remarkable differences in clinical presentation ranging from localized to highly metastatic. Although age and clinical stage are strong prognostic indicators, particular genetic aberrations, i.e. *MYCN *amplification and 17q gain, also have a profound predictive power [2,3]. Presently, three major clinico-genetic NB patient subgroups have been recognized (subgroup 1, 2A and 2B) [4]. Subgroup 1 consists of NB patients with favourable disease stage (stage 1, 2 and 4S), most often infants younger than one year of age presenting with tumours with a near triploid DNA content and a characteristic pattern of chromosomal instability including the consistent presence of an extra chromosome 17. The two other NB patient groups represent mainly older children with high-stage disease (stage 3 and 4) and poor prognosis. Both NB subgroups present with 17q-gain, but are distinguished by presence of *MYCN *amplification and 1p-deletion in subgroup 2B and 11q-deletion often in combination with 3p-deletion in subgroup 2A [3,5-9].

The first evidence for the occurrence of 11q-deletions in NB was obtained in 1991 [10]. However, it was not until recently that a specific patient subgroup with this particular genetic defect was recognized, representing approximately 20% of cases [5-9,11-13]. The recurrent finding of 11q-deletions in NB suggests the presence of a tumour suppressor gene residing on the long arm of chromosome 11. Additional functional evidence for this hypothesis came from the observation that differentiation of NB cells can be induced by transfer of an intact chromosome 11 into a NB cell line [14]. Although both comparative genomic hybridization (CGH) and loss of heterozygosity (LOH) studies indicate that the majority of the 11q-deletions are distal losses encompassing a large portion of the long arm [5-9,12,13,15,16], detection of rare small or interstitial deletions allowed the provisional localization of an SRO (shortest region of overlap) at 11q23.3 between markers D11S1340 and D11S1299, encompassing a distance of approximately 3 Mb [16]. When a single tumour with two small interstitial deletions is not taken into consideration, the SRO is defined by a small subset of tumours and spans 18 Mb between markers D11S898 and D11S1299 (according to UCSC Genome Browser, freeze version July 2003). This region harbours *SDHD*, which encodes the small subunit D (cybS, cytochrome b558) of the mitochondrial respiratory chain complex II (succinate-ubiquinone oxidoreductase) [17,18] and was recently recognized as a prototype tumour suppressor gene [19].

The first evidence for a role of *SDHD *in tumour development was obtained by the discovery of germline mutations in this gene as the cause for familial paraganglioma (PGL) [19]. Somatic and occult germline *SDHD *mutations were also detected in patients with apparently sporadic pheochromocytoma (PC) [20,21]. It seems that most of the individuals with PC possess *SDHD *mutations in the 5' portion of the gene causing complete disassembly of complex II, whereas PGL are associated with mutations in the 3' region of the gene causing partial inactivation of its catalytic activity [19-28]. PGL and PC are histologically related to NB as they are all neural crest derived. NBs consist of immature neuroblasts, whereas PGL and PC contain mature chromaffin cells. Of further interest is the fact that, in addition to the well established role of *SDHD *in oxidative phosphorylation, *SDHD *has also been presumed to contribute to the function of the mitochondria as oxygen sensors. It was shown that *SDHD *inactivation leads to a pseudo-hypoxic state and upregulation of hypoxia responsive genes, possibly through increased production of reactive oxygen species (ROS) [23]. A hypoxia-induced shift toward a neural crest-like phenotype has been shown to result in more aggressive NB cells with increased potential to metastasize [29]. Consequently, inactivating *SDHD *mutations or reduced activity of *SDHD *might lead to impaired oxidative phosphorylation and hypoxia and thus contribute to NB oncogenesis.

In view of the above, we considered *SDHD *as a positional and functional candidate for the presumed NB tumour suppressor gene on 11q23. In order to search for evidence for involvement of *SDHD *in NB development, an extensive series of investigations was performed on the DNA, RNA and protein level.

## Methods

### NB patient and cell line samples

Neuroblastoma (NB) tumour samples (at least 70% tumour cells) were collected at the Ghent University Hospital (Ghent, Belgium) (n = 32) and in the Molecular Oncology Unit (Lyon, France) (n = 35). Ethical approval was obtained for the collection of the tumour samples. The latter group includes selected patients with stage 3 or 4 NB without *MYCN *amplification. For all NB patients constitutional leukocyte DNA was available. In addition, 31 NB cell lines were included in the analysis of which karyotypes were available. For 20 of these cell lines, comparative genomic hybridization (CGH) data and/or M-FISH (multicolour fluorescence in situ hybridization) results have been published [30-32]. For screening of sequence variants in a normal population, leukocyte DNA from 135 unrelated healthy individuals was used. DNA was extracted as previously described [33].

Cultures of NB cell lines N206, SK-N-AS, SK-N-SH, NMB, SK-N-FI, CLB-GA, LA-N-2 and NGP were treated with puromycine (100 μg/ml) during 6 hours in order to prevent possible nonsense mediated RNA decay of variant *SDHD *transcripts. RNA of the cell line pellets (treated and untreated) was extracted with the RNeasy Mini kit (Qiagen) according to the manufacturer, followed by RNase free DNase treatment on column (Qiagen). A fraction of the untreated NB cell line cultures was also used for functional enzyme assays.

### 11q23 status of samples

#### Determination of the 11q23 status in NB cell lines and tumours with LOH or FISH

The 11q status of the cell lines was evaluated using FISH. FISH was performed using the LSI *MLL *(11q23.3) SpectrumOrange probe (Vysis) and BAC clone RP11-93E4 for the *CRTAM *gene (11q24.1) in combination with a centromeric probe for chromosome 11. Labelling and FISH was performed as described [34]. For each case at least twenty metaphase chromosomes and 100 interphase nuclei were screened.

All NB patients were analyzed with 4 microsatellite markers on 11q23: D11S1986 (11q23.1), D11S1998 (11q23.3), D11S1356 (11q23.3) and D11S1299 (11q23.3), of which D11S1986 and D11S1998 are immediately flanking the *SDHD *gene. In order to discriminate between whole chromosome loss, and unbalanced 11q loss (= partial 11q loss), two microsatellite markers on 11p (D11S922 on 11p15.5 and D11S1324 on 11p14.1) were analyzed in patients that showed allelic imbalance for all 11q markers (positions of the markers are according to the UCSC Genome Browser, freeze version July 2003). Scoring of loss of heterozygosity (LOH) was performed by calculation of the allelic imbalance factor (AIF) [35], whereby AIF > 2 denotes allelic imbalance, and AIF > 5 denotes LOH. Experimental conditions for the fluorescent based LOH screening can be obtained from the authors upon request.

#### Homozygous deletion screening in NB cell lines

Real-time quantitative PCR primers were designed in the four exons of *SDHD *using Primer Express v2.0 (Applied Biosystems) (Table 1). Exon 1 was too small for primer design in the exonic region; therefore primers flanking the exonic region were designed. Real-time quantitative PCR and quantification was performed as described [33].

### MSP

On the 31 NB cell lines and on another series of 50 NB tumours of which 15 were included in the mutation analysis, methylation-specific PCR (MSP) was performed as described, with minor modifications [36]. MSP primers were designed using the web-based MSP design software MethPrimer [37] and checked for specificity using the methBLAST software  (Pattyn et al., in preparation). Primers were designed in a CpG island close to the start of the gene (putative *SDHD *promotor) (chr11: 111495002–111495330: UCSC Genome Browser freeze version July 2003) (methylated forward 5'GTAGTCGGGATCGAGTATTAGTGAGTC3', methylated reverse 5'AATAAACCGAAAATCGAAAAACGAT3', unmethylated forward 5'AGTTGGGATTGAGTATTAGTGAGTTGT3', unmethylated reverse 5'ACTAAATAAACCAAAAATCAAAAAACAAT3'). Amplification mixtures (50 μl) for the PCR reaction contained 50 ng template DNA, 1× Platinum Taq PCR reaction buffer (Invitrogen), 6 mM MgCl_2_, 200 μM of each dNTP, 1.25 U Platinum Taq polymerase (Invitrogen), 3% DMSO and 300 nM of each primer. The cycling conditions comprised 4 min polymerase activation at 93°C, 40 cycles with denaturation at 93°C for 30 sec, annealing at 64°C (methylated primers) or 65°C (unmethylated primers) for 30 sec and extension at 72°C for 30 sec, and a final extension for 5 min at 72°C. SssI methylase (New England Biolabs) treated DNA, following the manufacturer's instructions and normal human genomic DNA were used as a positive and negative control respectively after bisulfite modification.

### Mutation analysis

#### DHPLC analysis

Intronic primers flanking the *SDHD *exons were designed using Primer Express v2.0 (Applied Biosystems), based on the publicly available *SDHD *genomic sequence (accession number AB026906) (Table 2). PCR reactions were performed on a PTC-200 DNA engine (MJ Research). Amplification mixtures (25 μl) contained 10 ng template DNA, 1× Platinum Taq PCR reaction buffer (Invitrogen), 2.5 mM MgCl_2_, 200 μM of each dNTP, 1 U Platinum Taq polymerase (Invitrogen) and 500 nM of each primer. The cycling conditions comprised 3 min polymerase activation at 94°C, 35 cycles with denaturation at 92°C for 20 sec, annealing at 60°C for 20 sec and extension at 72°C for 2 min, a final extension for 5 min at 72°C and a slow decrease in temperature to 25°C over 30 minutes. One μl of the PCR products was analyzed on a Ready-To-Run Agarose Gel (1.2%) (Amersham Biosciences).

Denaturing high-pressure liquid chromatography (DHPLC) was performed using the Wave system (Transgenomic). The melting profile of each fragment was determined using the Wavemaker software v4.1 (Transgenomic). Crude PCR product was injected into a preheated, fully equilibrated chromatographic column for the DHPLC analysis. Exon 1 fragments were eluted at a temperature of T_m_(= 62.1°C)+0.7°C and T_m_+1.5°C. Exon 2 fragments were eluted at a temperature of T_m_(= 55.7°C)+4.8°C. Exon 3 fragments were eluted at a temperature of T_m_(= 57.4°C)-0.4°C, T_m_+1.1°C and T_m_+3.8°C. Exon 4 fragments were eluted at a temperature of T_m_(= 56.4°C)-0.4°C, T_m_+1.6°C and T_m_+3.2°C. Elution of the fragments was performed using standard conditions according to the manufacturer. Elution profiles were analyzed using the Wavemaker software.

#### Sequencing

Sequencing was performed on all cell lines and on tumour samples with aberrant DHPLC elution peaks (except from the noncoding region of exon 4 that was sequenced in all NB cell lines without preceding DHPLC mutation screening).

Amplified fragments were purified using the Montage PCR96 filter plates (Millipore) or by excision of the fragment of interest from a 1.5% TBE-agarose gel and purification on a GenElute Minus EtBr Spin Column (Sigma-Aldrich). Cycle sequencing was performed using purified amplicons (3–10 ng), the above-mentioned primers (Table 2) at a concentration of 80 nM and the ABI PRISM BigDye Terminators v3.0 Cycle Sequencing Kit (Applied Biosystems), with the following thermocycling conditions: 25 cycles at 92°C for 10 sec, 55°C for 5 sec and 60°C for 3.5 min. The products were run on an automated sequencer ABI3100 (Applied Biosystems) after isopropanol precipitation. Sequence analysis was performed with the SeqScape v1.1 software (Applied Biosystems).

#### Allelic discrimination screening for 2 sequence variants using MGB probes

PCR primers and minor groove binder (MGB) probes for sequence variant IVS4-32T>C were designed using Primer Express v2.0 following the user bulletin guidelines for the design of MGB probes (Applied Biosystems): forward primer 5'TTTTTTGCAGCCAAGTTATCTGTATAG3', reverse primer 5'TGTCCAAGGCCCCTAAAGAA3', MGB probe allele 1 5'TGTGGTTTTTtATTGATG3' labelled with 6-FAM and MGB probe allele 2 5'TGTGGTTTTTcATTGAT3' labelled with VIC. To address the frequency of the sequence variant g.7876A>G (Y93C) in a normal population, the following primers and probe were designed: forward primer 5'GGCTGCTTATTTGAATCCTTGCT3', reverse primer 5'ACTTGCCAGTGACCATGAAGAGT3' and MGB probe variant allele 5'ATGGACTgTTCCCTG3' labelled with VIC. The reaction mixture contained 10 ng of DNA, 100 nM of each MGB probe, 300 nM of each primer and 1× qPCR Mastermix (Eurogentec). For the screening of the g.7876A>G variant, multiplex PCR was performed using primers and probe of the normal allele of the above-mentioned SNP (IVS4-32T>C) and primers and probe for the variant allele g.7876A>G. Reactions were performed on the iCycler Thermal Cycler (Bio-Rad) with the following thermocycling conditions: an initial activation step at 95°C for 10 min, 50 cycles of 95°C for 15 sec and 60°C for 1 min. Allelic discrimination data analysis was performed on the iCycler IQ Optical System Software v3.0a (Bio-Rad).

The SNP info for the IVS4-32T>C variant was submitted to NCBI's SNP database (sn#5606973, *SDHD*_IVS4-32).

### Full-length SDHD mRNA amplification

In order to investigate predicted or putative splice variants caused by the 4 bp-deletion in NB cell line N206 and *SDHD *sequence variants present in other cell lines, the coding region of the full-length *SDHD *mRNA was amplified for cell lines N206, SK-N-AS, SK-N-SH, NMB, SK-N-FI, CLB-GA, LA-N-2 and NGP, before and after puromycin treatment. RNA extraction, DNase treatment and cDNA synthesis were performed as described [38]. Subsequent PCR was performed with forward primer 5'AGGAACGAGATGGCGGTTCTC3' (exon 1) and reverse primer 5'GCTTCCACAGCATGGCAACA3' (exon 4). PCR products were run on an agarose gel, purified and sequenced using the above-mentioned protocol. The sequence information also provided evidence on the allelic mRNA expression status of *SDHD*.

### Quantification of SDHD expression using real-time quantitative RT-PCR

Relative *SDHD *expression levels were determined using an optimized two-step SYBR Green I RT-PCR assay [38] with minor modifications in 31 cell lines, 7 normal control samples (human brain, trachea, lung, heart, breast, kidney, liver) and in laser capture microdissected foetal neuroblast cells [39]. The comparative C_T _method was used for quantification. PCR reagents were obtained from Eurogentec as SYBR Green I mastermixes and used according to the manufacturer's instructions. Primers in exon 3 were designed using Primer Express (see Primers Exon 3 in Table 1). Reactions were run on an ABI5700 (Applied Biosystems). Gene expression levels were normalized using the geometric mean of the 4 most stable internal control genes in NB (i.e. *UBC*, *HPRT1*, *SDHA *and *GAPD*) as reported previously [40].

### Complex II activity and protein assays

Enzyme activities were determined spectrophotometrically as previously described [41].

Protein amount was determined with immunoblot analysis of complex II Fp fragment as previously described [42]. Relative protein amounts of complex II compared to complex IV were measured using the TotalLab software (Amersham Biosciences).

### Ultrastructural analysis of mitochondria in NB cell lines

Mitochondria of NB cell lines LA-N-2, SK-N-AS, CLB-GA, NGP, CHP-901, SK-N-SH, SK-N-FI, N206, SJNB-12, SJNB-8, NMB, SJNB-10 and IMR-32, breast carcinoma cell line MCF-7 and, Ewing sarcoma cell line SK-N-MC were analysed by electron microscopy. Monolayers from these cell lines were briefly rinsed twice in PBS and then immersed at room temperature in 3 % glutaraldehyde buffered with Na-cacodylate at pH 7.3 for 1 h. After rinses in this buffer with 1 % bovine serum albumin, cells were scraped off with a rubber policeman, and centrifuged with 3 % glutaraldehyde. After washing, the pellets were postfixed in 2 % buffered OsO_4 _for 1 h at 4°C. Block staining in uranylacetate (UAc) in 70 % ethanol was followed by dehydration in ethanol and propylene oxide, and embedding in Epon. Ultrathin sections were counterstained with UAc and lead. At least 2 cells in each culture were photographed at a magnification of 20,000 in a Zeiss electron microscope operating at 50 KV. Per culture, 25–63 mitochondria were examined. Their projected length (largest straight distance) was measured and matrix electron density was compared to the surrounding cytosol.

Electron microscopic files were made on 11 archived neuroblastic tumours. In addition, previously published cases were examined for mitochondria morphology.

## Results

### SDHD deletion, mutation and methylation analysis

#### 11q23-deletion screening

Previous karyotyping, comparative genomic hybridization (CGH) and/or M-FISH revealed 11q-deletions in 9 out of 31 neuroblastoma (NB) cell lines (CLB-GA, GI-ME-N, IMR-32, LA-N-6, NBL-S, NGP, SK-N-AS, NMB, N206) [30-32]. In this study, the presence of 11q23-deletions was confirmed by FISH in all these cell lines except N206 for which the deletion was located distal to the *MLL *locus (11q23.23) (not shown) (Table 3). Sequencing analysis of *SDHD *on 11q23 demonstrated that the allelic imbalance in NMB does not cause loss of heterozygosity (see later). No previously unnoticed submicroscopic 11q23-deletions were detected. Screening for homozygous deletions in all *SDHD *exons was negative for the 31 NB cell lines.

In 20 of the 67 NB tumour samples, loss of heterozygosity (LOH) or allelic imbalance (AI) (AIF > 2) in the 11q23 region was found (Table 3): unbalanced 11q LOH (i.e. partial allelic loss of the long arm of chromosome 11) in 2/32 patients of the Ghent University Hospital (Ghent, Belgium) and in 7/35 patients of the Molecular Oncology Unit (Lyon, France) and loss of markers on both chromosome arms (indicating whole chromosome 11 loss, or co-occurrence of 11q and 11p allelic loss) in 3/32 patients of the Ghent University Hospital and 8/35 patients of the Molecular Oncology Unit (Table 3). The higher frequency of chromosome 11 LOH in the patient subgroup of the Molecular Oncology Unit can be explained by the selection for patient samples of high stage without *MYCN *amplification, in contrast to the other patient subgroup for which samples were unselected.

#### Mutation analysis

Denaturing high performance liquid chromatography (DHPLC) analysis and subsequent sequencing of the *SDHD *gene in 31 NB cell lines and 67 NB tumour samples revealed the presence of sequence variants in 5 NB cell lines and 4 NB tumour samples (Table 4).

**Figure 1:**
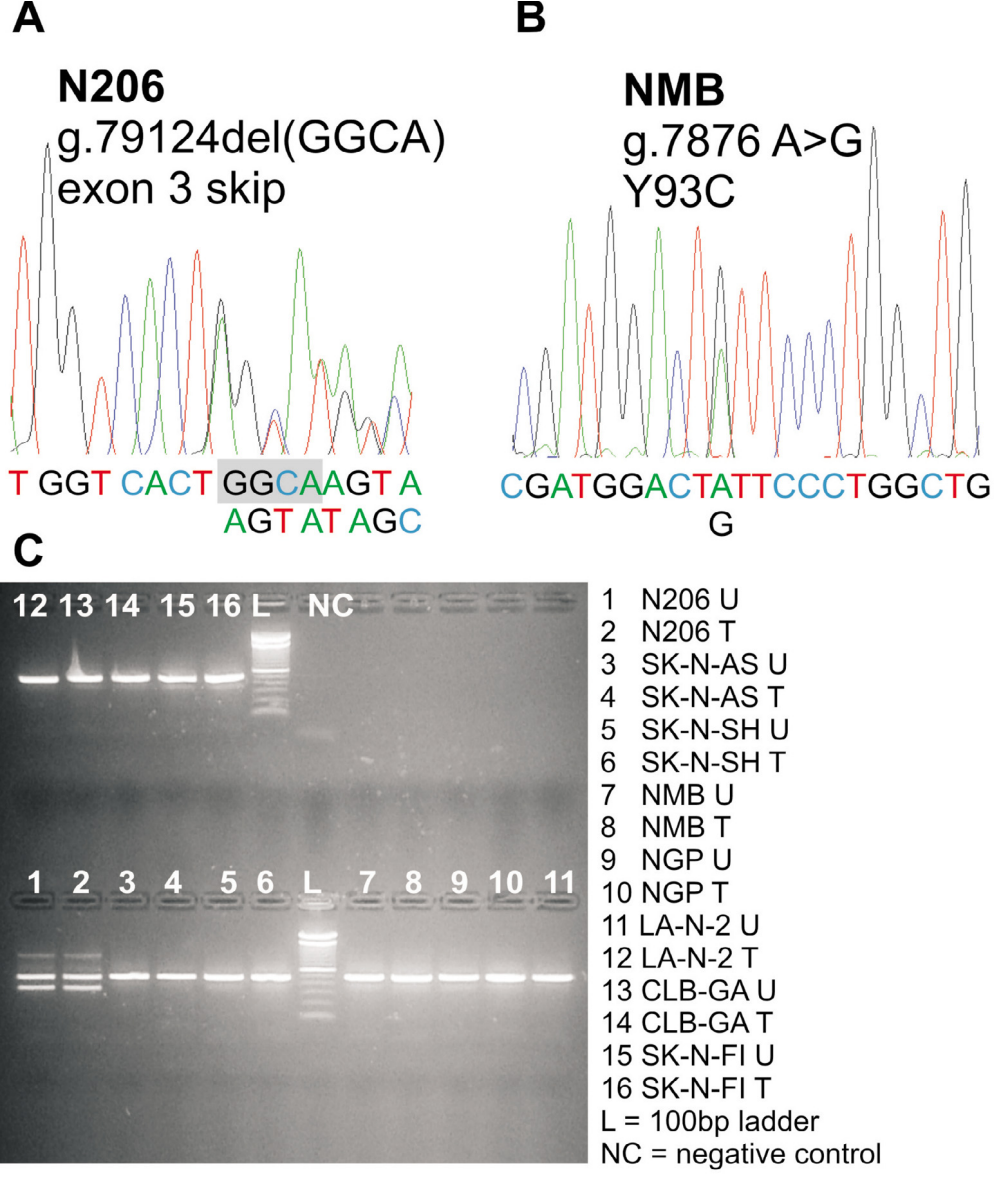
Details of sequencing profiles: (A) Deletion of GGCA in cell line N206 causing skip of exon 3 and (B) Y93C mutation in cell line NMB; (C) RT-PCR (reverse transcriptase PCR) on cell lines grown with or without puromycine (T = treated, U = untreated) revealed a transcript variant in cell line N206, caused by the GGCA deletion (lane 1 and 2).

Two variants were considered as bona fide mutations (Figure 1). The first, a Y93C missense mutation in cell line NMB, was not detected in 135 unrelated healthy individuals. The second variant detected in NB cell line N206, represented a 4 bp deletion on the exon-intron boundary causing an exon 3 skip leading to a premature stop codon. Interestingly, both effects are located within regions that are frequently affected in paraganglioma (PGL). Unfortunately no normal or primary tumour material of the patients from which the N206 and NMB cell lines were derived was available to test whether these are germline or somatic mutations.

In one patient without 11q allelic loss (F11) we observed in both tumour and constitutional DNA a TCTA insertion at position IVS2+37. However, no additional tumour material nor parental material was available for further analysis. So, it remains unclear whether this is a true mutation or a rare polymorphism.

In addition, 1 new and 4 known polymorphisms were observed. The H50R variant found in cell line LA-N-2 was described as a polymorphism in several studies [43-45]. This is also true for the G12S change found in tumour and constitutional DNA of patient F18 [21]. The previously reported polymorphisms IVS3-29A>G [25] and S68S [25,27,28,44,46,47] were detected in cell lines NGP, NMB and SK-N-FI, in both tumour and constitutional DNA of patients F18 and F35, and in constitutional DNA of patient F22. In all cases, these last two polymorphism (IVS3-29A>G and S68S) were present together with the IVS4-32T>C variant, previously described by Taschner and colleagues [28]. Allelic discrimination screening in 135 unrelated individuals revealed an incidence of the IVS4-32T>C polymorphism of 4.4% (= 6/135; allele frequency 2.2%). This is similar to the incidence found in NB cell lines (3/31 = 9.7%) and NB patient constitutional DNA (3/67 = 4.5%, allele frequency = 2.2%).

The presence of the IVS3-29A>G, S68S and IVS4-32T>C variants in a cell line (NGP) and two tumours (F18 and F35), in which one of both *SDHD *alleles has been deleted, indicates that all three variants are located on the same allele, representing a low frequent haplotype.

#### MSP analysis

*SDHD *promotor hypermethylation was tested for 31 NB cell lines and 50 NB patients using methylation-specific PCR (MSP). No evidence for methylation was obtained in any of the analyzed NB cases.

#### Analysis of the 4 bp deletion in the cell line N-206

Amplification of the full-length *SDHD *cDNA showed that a 4 bp deletion in the intron-exon boundary in cell line N206 caused skipping of exon 3 leading to a premature stop codon.

No alternative transcripts could be detected in cell lines NMB, SK-N-FI, NGP and LA-N-2 carrying basepair variants (and 3 control cell lines without sequence variants SK-N-SH, SK-N-AS and CLB-GA) when grown with or without puromycin (Figure 1).

The above-mentioned cDNA transcript sequencing revealed that *SDHD *is bi-allelically expressed, thus supporting recent observations in lymphoblastoid cell lines, adult kidney and adult and fetal brain [19,22], but in contrast with the initially reported paternal mono-allelic expression in PGL tissue [22].

### SDHD mRNA expression analysis

**Figure 2:**
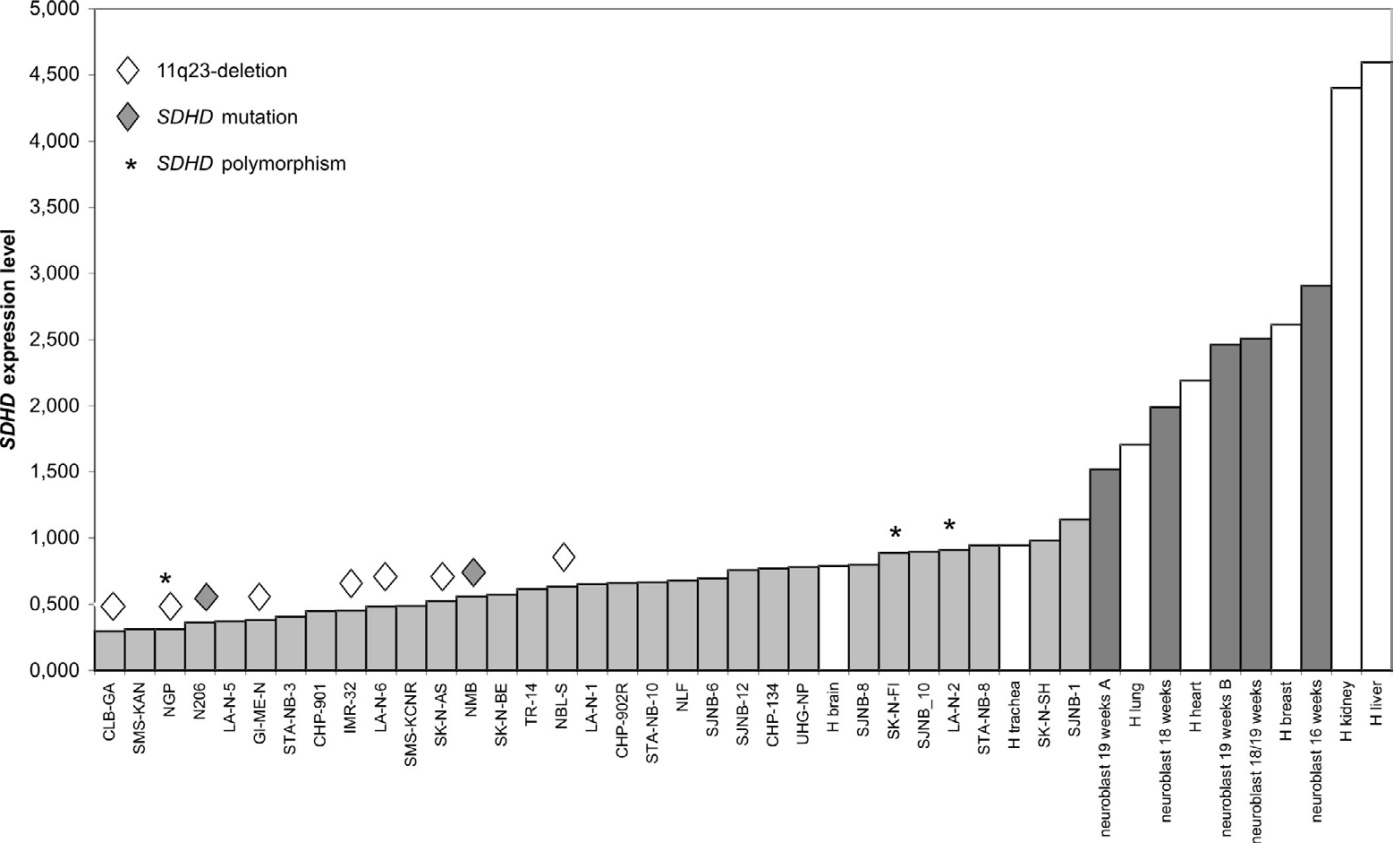
*SDHD *mRNA levels in NB cell lines (light gray), neuroblast cells (gray) and human normal control samples (white). Significantly reduced *SDHD *mRNA expression levels in NB cell lines with 11q23 allelic loss compared to 11q23 intact NB cell lines (P = 5.31E-06) and normal tissue samples (P = 1.49E-05).

*SDHD *expression levels were measured using real-time quantitative PCR in 31 NB cell lines, normal foetal neuroblast cells (16, 18 and 19 weeks gestational time) and 7 normal adult tissues (brain, heart, kidney, liver, lung, trachea and breast) (Figure 2). The *SDHD *mRNA level was significantly lower in NB cell lines compared to both normal neuroblast cells (Mann-Whitney test: P = 5.31E-06) and normal adult tissue mRNA samples (Mann-Whitney test: P = 1.49E-05).

*SDHD *mRNA levels were significantly reduced in cell lines with 11q allelic loss and *SDHD *mutated cell lines (i.e. NMB and N206) (N = 9) compared to cell lines without 11q allelic loss (N = 22) (Mann-Whitney test: P = 1.49E-03).

### SDHD functional analysis

As the *SDHD *gene encodes the small subunit D of the mitochondrial respiratory chain complex II we decided to assess the effect of the basepair variants on the activity of complex II of the respiratory chain by spectrophotometrical measurements in 5 NB cell lines (N206, NMB, SK-N-FI, NGP and LA-N-2) and 3 control NB cell lines without sequence variants (SK-N-SH, SK-N-AS and CLB-GA). No significant differences in complex II enzyme activity could be demonstrated. Although, in LA-N-2 a slight decrease of complex II activity was observed (data not shown).

On above-mentioned cell lines and NB cell lines CHP-901, SJNB-12, SJNB-8, SJNB-10 and IMR-32, breast cancer cell line MCF7 and Ewing sarcoma cell line SK-N-MC, immunoblotting of the Fp fragment of complex II showed no significant variation in abundance among the tumour cell lines (data not shown).

### Ultrastructural morphology of mitochondria in NB cell lines

**Figure 3:**
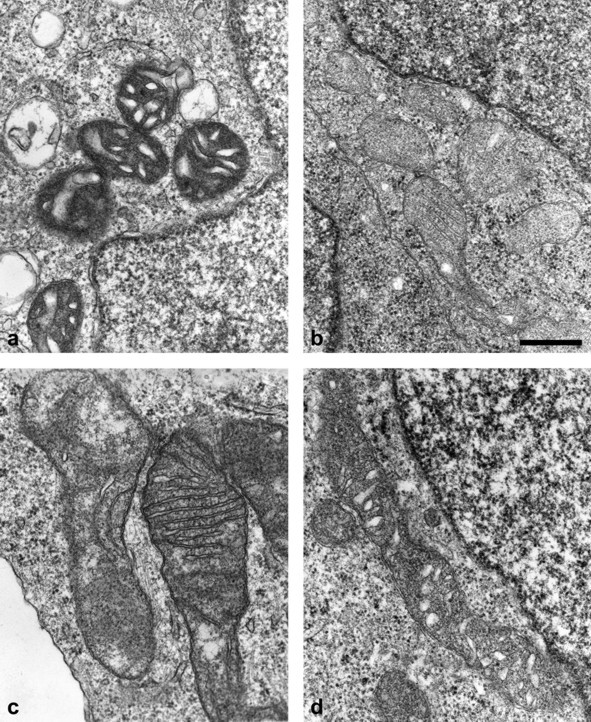
Mitochondrial ultrastructure shows heterogeneity between cell lines (same final magnification for the 4 images, marker = 0.5 μm): (A) NB cell line N206: dilated crista spaces in small mitochondria with a dense matrix; (B) NB cell line NMB: small mitochondria with narrow cristae and light matrix, so-called orthodox configuration, (C) NB cell line SJNB-8: unusually large mitochondria in orthodox configuration (narrow cristae), some areas in the matrix are cleared and lack cristae; (D) NB cell line LA-N-2: very large mitochondria with dilated cristae and dense matrix.

Electron microscopic analysis of NB cell lines revealed that the morphology of the mitochondria is heterogeneous between the different cell lines, with respect to length, dilated intracrista spaces and condensation of the matrix (Table 5 and Figure 3). In most of the cell lines the electron dense matrix granules are absent. A striking observation are dilations of the mitochondrial intracrista spaces in most of the NB cell lines including N206 (Figure 3A), but not NMB (Figure 3B). Cell line LA-N-2 shows very large mitochondria (Figure 3D). However, these observations are not the same as described for PGL, where swollen mitochondria are seen with an empty matrix and short or absent cristae [48].

In order to examine whether dilated mitochondrial cristae are a feature of many, or all NBs, we studied the mitochondria in electron micrographs from 11 archived and 22 previously published neuroblastic tumours [49-53]. Dilated cristae were seen in 11 tumours but they were limited to a minority of the mitochondria (2–23%), in contrast to several of the cell lines of which most mitochondria are altered. In several of the analyzed NB tumours, partially vacuolated matrices were occasionally observed.

## Discussion

In this study, we investigated the possible involvement of *SDHD *in neuroblastoma (NB) tumourigenesis. In a first step, mutation and methylation analyses were performed on a large panel of NB cell lines and tumours. A total of seven sequence variants (in nine different samples) were detected of which two could represent bona fide mutations, i.e. missense mutation Y93C in cell line NMB and a 4 bp deletion in cell line N206.

The Y93C sequence variant has not been reported previously and screening of 135 unrelated healthy individuals for this variant was negative. The substituted amino-acid is located within a region of the *SDHD *protein frequently altered due to germline mutations in paraganglioma (PGL) families (loss of Y93 [22] and two missense mutations, i.e. D92Y [19,28,46] and L95P [28]). These residues are part of the third transmembrane helix of the SDHD protein [54].

The second mutation has not been reported either. This mutation results from a 4 bp deletion in the 3' exon-intron boundary of exon 3 resulting in skipping of exon 3 leading to a transcript with a premature stop codon. The predicted truncated protein has another carboxyterminal amino-acid sequence from H56 on and its normal function is assumed to be impaired as carboxyterminal amino-acids involved in ubiquinone and heme b binding are missing (H71, D82 and Y83) and consequently the structure of the transmembrane subunit and/or association of the catalytic domain subunits SDHA and SDHB to the membrane would be disrupted [54].

The functional consequence of one sequence variant located within an intronic sequence (IVS2+37ins(TCTA)) is more difficult to evaluate due to lack of fresh tumour material, and parental DNA. Further analysis is needed in order to reveal a possible effect on splicing or RNA stability.

Finally, one new and 4 known polymorphisms were detected in 5 NB cell lines and 3 tumour samples. Additional screening for homozygous deletions in all cell lines and methylation in cell lines and tumours were negative.

Based upon these results, we can exclude a role for *SDHD *as a classical tumour suppressor gene in NB. However, the finding of two apparently bona fide *SDHD *mutations in NB without allelic loss of distal 11q leaves the possibility open that the gene contributes to NB oncogenesis due to haplo-insufficiency, rather than functional inactivation of both alleles. In order to investigate this possibility, we decided to perform further studies at transcript and protein level. Interestingly, *SDHD *expression was shown to be consistently lower in cell lines with 11q allelic loss versus NB cell lines without loss and also significantly decreased in NB cell lines as compared to normal foetal adrenal neuroblast cells of 16, 18 and 19 weeks gestational time with a mean fold difference of 3.61 between neuroblast cells and NB cell lines. A similar correlation between 11q LOH and reduced *SDHD *expression was recently described in colorectal and gastric cancer [55]. Our findings at mRNA transcript level, however, did not match with results obtained from further analysis at protein level. Complex II activity and quantitative protein analysis revealed no significant difference between cell lines with or without 11q allelic loss or *SDHD *mutation. However, measurement of complex II activity might only reflect part of the functional properties of SDHD. Also, measurement of differences in protein quantity is far less sensitive than Q-PCR at transcript levels. Therefore, these observations at present do not fully exclude *SDHD *involvement in NB. Finally, we also looked at the morphologic characteristics of the mitochondria as a possible clue to SDHD dysfunction. In keeping with, at best, partial loss of function of *SDHD*, we did not observe similar gross morphologic changes as reported for PGL with *SDHD *mutations (swelling with loss of matrix density and generalized rarefaction of cristae), the latter being characterized by destabilization of complex II with loss of enzymatic activity [48]. However, most of the cell lines showed dilated mitochondrial cristae. It has been demonstrated that this is a reversible phenomenon, and parallels arise in intracellular ADP/ATP ratio or low energy state [56]. Subsequent combined ultrastructural and biochemical studies from several authors indicated that dilation of cristae follows a decrease in mitochondrial membrane potential that can be provoked by various experimental procedures [57,58]. This configuration was detected in only a small percentage of mitochondria in archived sections of NB tumours and in sections published earlier. Morphologic analysis of mitochondria in NB thus far received little attention. The true significance of the observed mitochondrial morphological changes in NB is intriguing, but does not appear to be related to the mutations we have found.

## Conclusions

In contrast to previous findings in PGL and PC, this study excludes a classical two hit Knudson model for *SDHD *involvement in NB. However, the finding of, albeit rare, bona fide mutations and reduced expression of *SDHD *in NB with 11q allelic loss hints at a possible haplo-insufficient contribution to tumour development. A better understanding of the different functions of SDHD, in particular its possible contribution to energy independent apoptosis involving the release of cytochrome c and procaspases, will allow further functional assays to asses how this gene contributes to tumour development in general, and the high stage NB phenotype in particular [59,60].

Evidence for contribution to a cancer phenotype through haplo-insufficiency has recently been obtained for a number of loci, including *CDKN1B *(p27^Kip1^) [61,62], *TP53 *(p53) [63], *DMP1 *[64], *PTEN *[65], *APC *[66] and *NKX3.1 *[67]. In mouse models for some of these genes, loss or mutation of one allele increased tumour susceptibility despite expression of the remaining wild-type allele [68]. Although the present data on protein and functional level do not provide consistent evidence for the haplo-insufficient involvement of *SDHD *in NB, a bipartite mechanism as tumour suppressor gene for the *SDHD *gene, as described for the *APC *gene can at present not be fully excluded. Following this hypothesis, germline mutations in *SDHD *would predispose to PGL or PC development. Rare somatic mutations and more typically loss of one allele could contribute to the metastasizing NB tumour phenotype (and possible also other tumour types), not as an initiating step but rather as later event in tumour development. However, further evidence is needed to support the haplo-insufficient involvement of *SDHD *in cancer. Ultimately, knockout mice for the *SDHD *gene leading to haplo-insufficiency for *SDHD *in neuroblast progenitor cells, would be the appropriate test to evaluate this hypothesis.

## List of abbreviations

AIF = allelic imbalance factor

CGH = comparative genomic hybridization

DHPLC = denaturing high performance liquid chromatography

LOH = loss of heterozygosity

MGB = minor groove binder

MSP = methylation specific PCR

NB = neuroblastoma

PC = pheochromocytoma

PGL = paraganglioma

ROS = reactive oxygen species

*SDHD *= succinate dehydrogenase, subunit D

SNP = single nucleotide polymorphism

SRO = shortest region of overlap

## Competing interests

The authors declare that they have no competing interests.

## Author's contributions

KDP carried out the genomic and transcriptomic studies, and drafted the manuscript. JH performed the methylation studies. JS carried out the immunoblottings and spectrophotometric analysis that was evaluated by RVC. AN performed the ultrastructural analysis that was screened and discussed by CV, FR and MP. GL and NVR collected the tumour material. JV and FS participated in the study's design and coordination. All authors have reviewed the manuscript and FS and ADP were the final editors of the manuscript.

## Pre-publication history

The pre-publication history for this paper can be accessed here:



## Figures and Tables

**Table 1:** Primer sequences used for homozygous exon deletion screening with real-time quantitative PCR

***amplicon***	***forward***	***reverse***
***SDHD *exon 1**	5'AGGAACGAGATGGCGGTTCT3'	5'TCCTAGGGCACCGCAAAC3'
***SDHD *exon 2**	5'CCAGTGGTCAGACCTGCTCAT3'	5'TGCTGCACTCCACACCATTC3'
***SDHD *exon 3**	5'GACTAGCGAGAGGGTTGTCAGTGT3'	5'CATCGCAGAGCAAGGATTCA3'
***SDHD *exon 4**	5'TGGCACTTTCAGCTTTAACCTTT3'	5'CACAGCATGGCAACAGCTTT3'

**Table 2:** Primer sequences used for denaturing high performance liquid chromatography (DHPLC) and sequencing

***amplicon***	***forward***	***reverse***
***SDHD *exon 1**	5'GCACCGCCTCTCGACTTC3'	5'TGCTGTGATTTCGGTATTTTCTTC3'
***SDHD *exon 2**	5'AACCCCAGTGAAATAGATGCTATCTTC3'	5'AGTCCTGCTAAAGGCATGACCATTA3'
***SDHD *exon 3**	5'CACTGCCTGTCAGTTTGGGTTAC3'	5'GGGCATTTCAATCAACTTCTCCC3'
***SDHD *exon 4**	5'TCCCCTAAAGAAGCAAACAGTGAC3'	5'GAGCTTAATGGCATGACAAAGCAG3'
***SDHD exon 4 Noncoding region (only for sequencing)***	5'GTGGTTTTTTATTGATGTTATGATTTT3'	5'AATCTCAATTTACAGTTGGTAGTATTTT3'

**Table 3:** (A) 11q23 LOH data of NB patients (35 patients form the Molecular Oncology Unit (Lyon, France) (F-samples) and 32 patients from the Ghent University Hospital (Ghent, Belgium) (G-samples)). Based on the allelic imbalance factor (AIF) of 6 markers (4 on 11q23 and 2 on 11p) normal 11q23 status was distinguished from unbalanced 11q LOH and loss of both 11q and 11p (here indicated as whole chromosome loss) (- = no data available) and (B) 11q23 status in NB cell lines based on FISH, LOH, karyotypes, CGH and/or M-FISH

**A**									
**NB tumour case**	**11q23 LOH status**	***MYCN *ampl**	**1p del**	**stage**	**NB tumour case**	**11q23 LOH status**	***MYCN *ampl**	**1p del**	**stage**
**F1**	**normal**	no	no	3	**G1**	**normal**	no	no	4
**F2**	**normal**	no	no	3	**G2**	**whole chr11 loss**	no	no	4
**F3**	**normal**	no	no	3	**G3**	**normal**	no	no	1
**F4**	**normal**	no	no	4	**G4**	**normal**	no	no	1
**F5**	**whole chr11 loss**	no	no	4	**G5**	**normal**	no	no	2
**F6**	**unb [11q]LOH**	no	no	4	**G6**	**normal**	-	no	3
**F7**	**normal**	no	no	4	**G7**	**normal**	-	-	4
**F8**	**normal**	no	no	3	**G8**	**normal**	no	no	1
**F9**	**normal**	no	no	4	**G9**	**whole chr11 loss**	no	no	4
**F10**	**whole chr11 loss**	no	no	3	**G10**	**normal**	no	no	4
**F11**	**normal**	no	no	4	**G11**	**normal**	no	no	1
**F12**	**normal**	no	no	4	**G12**	**normal**	no	no	4
**F13**	**normal**	no	no	3	**G13**	**normal**	no	yes	1
**F14**	**whole chr11 loss**	no	no	3	**G14**	**whole chr11 loss**	no	no	3
**F15**	**whole chr11 loss**	no	yes	4	**G15**	**normal**	no	no	3
**F16**	**normal**	no	no	3	**G16**	**normal**	-	no	4
**F17**	**unb [11q]LOH**	no	no	4	**G17**	**normal**	no	no	3
**F18**	**unb [11q]LOH**	no	yes	4	**G18**	**normal**	no	yes	1
**F19**	**normal**	no	no	4	**G19**	**normal**	no	no	4S
**F20**	**normal**	no	no	3	**G20**	**normal**	yes	yes	3
**F21**	**unb [11q]LOH**	no	no	4	**G21**	**unb [11q]LOH**	no	no	3
**F22**	**whole chr11 loss**	no	no	3	**G22**	**unb [11q]LOH**	yes	yes	4
**F23**	**normal**	no	no	3	**G23**	**normal**	no	no	4
**F24**	**normal**	no	no	3	**G24**	**normal**	yes	yes	4
**F25**	**whole chr11 loss**	no	no	3	**G25**	**normal**	no	no	4
**F26**	**unb [11q]LOH**	no	no	4	**G26**	**normal**	no	yes	4
**F27**	**whole chr11 loss**	no	no	3	**G27**	**normal**	no	no	3
**F28**	**normal**	no	no	3	**G28**	**normal**	-	no	2
**F29**	**unb [11q]LOH**	no	no	4	**G29**	**normal**	no	no	3
**F30**	**normal**	no	yes	4	**G30**	**normal**	no	no	1
**F31**	**normal**	no	no	4	**G31**	**normal**	-	no	3
**F32**	**normal**	no	no	4	**G32**	**normal**	no	no	4S
**F33**	**whole chr11 loss**	no	no	3					
**F34**	**normal**	no	yes	4					
**F35**	**unb [11q]LOH**	no	no	4					
**B**									
**NB cell line**	**11q23 status**	***MYCN *ampl**	**1p del**						
**CHP-134**	**normal**	yes	yes						
**CHP-901**	**normal**	yes	yes						
**CHP-902R**	**normal**	yes	yes						
**CLB-GA**	**deletion**	no	no						
**GI-M-EN**	**deletion**	no	yes						
**IMR-32**	**deletion**	yes	yes						
**LA-N-1**	**normal**	yes	yes						
**LA-N-2**	**normal**	yes	no						
**LA-N-5**	**normal**	yes	yes						
**LA-N-6**	**deletion**	no	yes						
**N206**	**normal**	yes	yes						
**NBL-S**	**deletion**	no	no						
**NGP**	**deletion**	yes	yes						
**NLF**	**normal**	yes	yes						
**NMB**	**normal**	yes	yes						
**SJNB-12**	**normal**	no	yes						
**SJNB-1**	**normal**	no	yes						
**SJNB-10**	**normal**	yes	yes						
**SJNB-6**	**normal**	yes	yes						
**SJNB-8**	**normal**	yes	yes						
**SK-N-AS**	**deletion**	no	yes						
**SK-N-BE**	**normal**	yes	yes						
**SK-N-FI**	**normal**	no	no						
**SK-N-SH**	**normal**	no	no						
**SMS-KAN**	**normal**	yes	yes						
**SMS-KCNR**	**normal**	yes	yes						
**STA-NB-10**	**normal**	yes	yes						
**STA-NB-3**	**normal**	yes	yes						
**STA-NB-8**	**normal**	yes	yes						
**TR-14**	**normal**	yes	yes						
**UHG-NP**	**normal**	yes	yes						

**Table 4:** *SDHD *base pair variants found in NB tumour samples and cell lines, the position of the variant, the change in the protein caused by the variant and the conclusion (mutation or polymorphism); also listed for each tumour and cell line are the genomic status for chromosome arm 11q (n.d. = not done) (for cell lines: chromosome 11 centromere copy number versus 11q23 copy number according to FISH, for tumours: normal, unbalanced LOH (unb [11q]LOH) or whole chromosome 11 loss according to microsatellite marker analysis), *MYCN *status (normal or amplified), 1p status and tumour stage when available (- = not available).

**NB tumour case number / NB cell line**	**11q23 status by microsatellite marker analysis**	11q23 status by FISH	***SDHD *base pair variant**	**exon**	**change in protein**	**mutation (M) or polymorphism (P)**	**variant present in constitutional (C)/ tumour (T)**	***MYCN***	**1p del**	**stage**
**F11**	**normal**	n.d.	g.6911ins(TCTA)	IVS2+37ins(TCTA)	? (see text)	? (see text)	C+T	normal	no	4
**F18**	**unb [11q] LOH**	n.d.	g.5842 G>A	1	G12S	P	C+T	normal	yes	4
			g.7750 A>G	IVS3-29 A>G		P	C+T			
			g.7802 C>T	3	S68S	P	C+T			
			g.13678 T>C	IVS4-32 T>C		P	C+T			
**F22**	**whole chr11 loss**	n.d.	g.7750 A>G	IVS3-29 A>G		P	C	normal	no	3
			g.7802 C>T	3	S68S	P	C			
			g.13678 T>C	IVS4-32 T>C		P	C			
**F35**	**unb [11q] LOH**	n.d.	g.7750 A>G	IVS3-29 A>G		P	C+T			
			g.7802 C>T	3	S68S	P	C+T			
			g.13678 T>C	IVS4-32 T>C		P	C+T			
**LA-N-2**	n.d.	**2/2 heterozygous**	g.6854 A>G	2	H50R	P	-	amplified	no	4
**N206**	n.d.	**2/2 heterozygous**	g.79124del(GGCA)	3	exon 3 skip, premature stop codon	M	-	amplified	yes	4
**NGP**	n.d.	**2/1 hemizygous**	g.7750 A>G	IVS3-29 A>G		P	-	amplified	yes	-
			g.7802 C>T	3	S68S	P				
			g.13678 T>C	IVS4-32 T>C		P				
**NMB**	n.d.	**4/3 allelic imbalance/ heterozygous (based on sequence)**	g.7750 A>G	IVS3-29 A>G		P	-	amplified	yes	4
			g.7802 C>T	3	S68S	P				
			g.7876 A>G	3	Y93C	M				
			g.13678 T>C	IVS4-32 T>C		P				
**SK-N-FI**	n.d.	**2/2 heterozygous**	g.7750 A>G	IVS3-29 A>G		P	-	no	no	-
			g.7802 C>T	3	S68S	P				
			g.13678 T>C	IVS4-32 T>C		P				

**Table 5:** Ultrastructural analysis of mitochondria

	dilation of cristae*	**density of matrix°**	**configuration#**	**matrix granules°°**	**mean projected length (μm)**	**max projected length (μm)**
CHP-901	+	-+	+	+	0,65	1,31

CLB-GA	++	+		+	0,69	1,92

IMR-32	+	+	+	-	0,69	1,27

LA-N-2	++	+		-	0,82	2,18

MCF-7	-	+	+	-	1,06	3,11

N-206	+++	+		-	0,84	1,65

NGP	+++	+		-	0,84	1,74

NMB	-	-	+	-	0,68	1,26

SJNB-10	+++	+		-	0,63	1,69

SJNB-12	+	+		-	0,47	1,23

SJNB-8	-	-+	+	-	1,02	3,40

SK-N-AS	++	+		-	0,66	1,47

SK-N-FI	+	-	+	-	0,65	1,32

SK-N-MC	-	-	+	-	0,90	2,03

SK-N-SH	+	-+	+	+	0,56	1,31

*: +cristae of some mitochondria dilated; ++cristae of many mitochondria dilated; +++all mitochondria have dilated cristae;

°: +matrix of most mitochondria is more electron dense than the cytosol; -+part of the mitochondria are dense, but others have a light matrix; -mitochondrial matrix is lighter than or equal to cytosol;

#: +mitochondria with an orthodox configuration are present; in the same cell or culture other mitochondria may have dilated cristae and a dense matrix;

°°: -few or no matrix granules; +matrix granules visible in many or most mitochondria, normal image;
